# Supplementary material for: DOS-3 mediates cell-non-autonomous DAF-16/FOXO activity in antagonizing age-related loss of C. elegans germline stem/progenitor cells
Source: Nat Commun. 2024 Jun 8;15:4904. doi: 10.1038/s41467-024-49318-6 (PMC11162419; doi:10.1038/s41467-024-49318-6)
Supplement: Supplementary file 1 — Supplementary Information [file 41467_2024_49318_MOESM1_ESM.pdf]

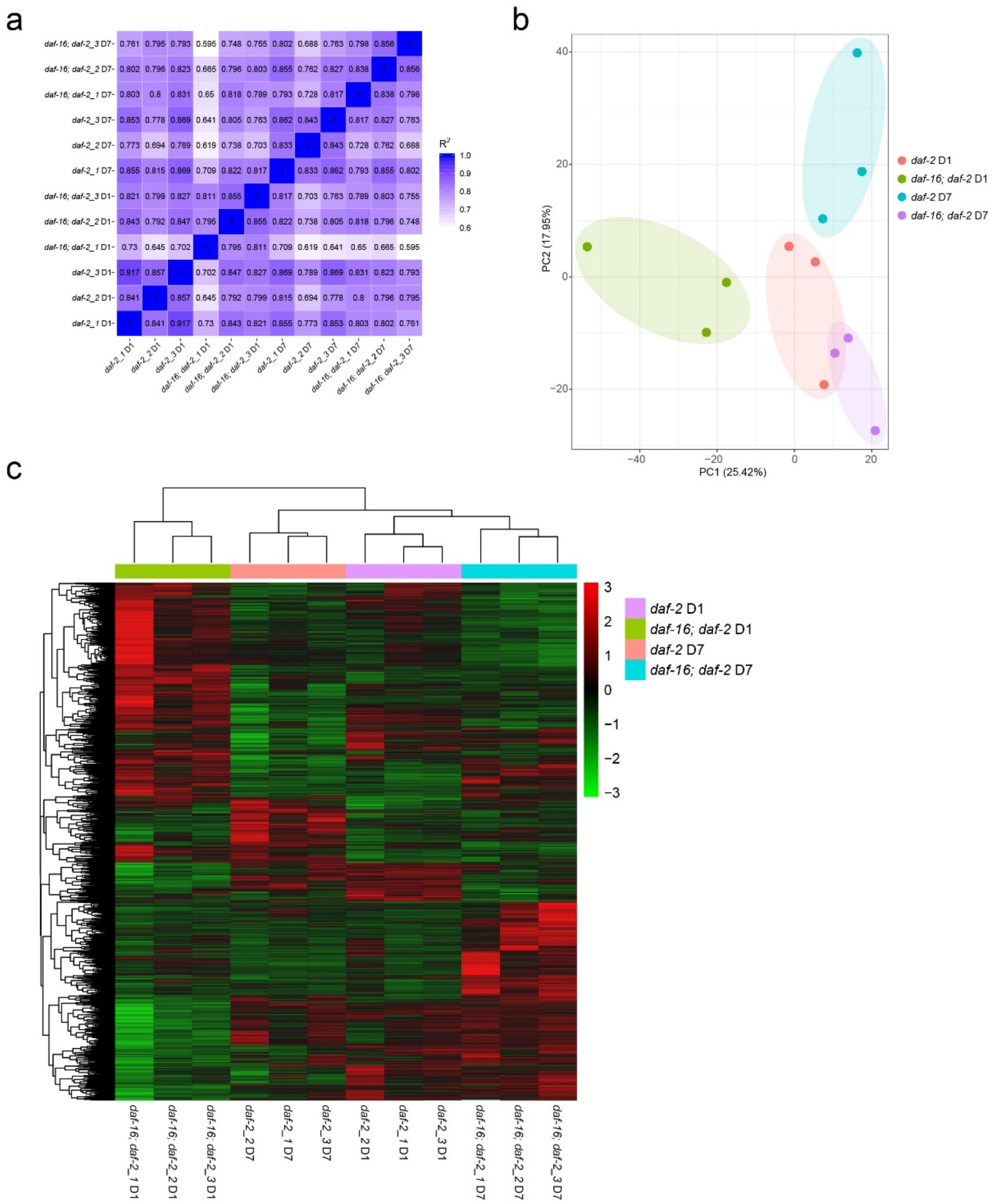

Supplementary Figure 1. RNA-seq transcriptional profiling of isolated adult PSG cells from *daf-2(rf)* and *daf-16(0); daf-2(rf)* worms, Related to Figure 1. a, Pearson correlation of young (D1) and aging (D7) PSG samples from *daf-2(rf)* and *daf-16(0); daf-2(rf)* animals. **b,** Principal component analysis of samples obtained for this study. **c,** Heat map of gene expression in our samples.

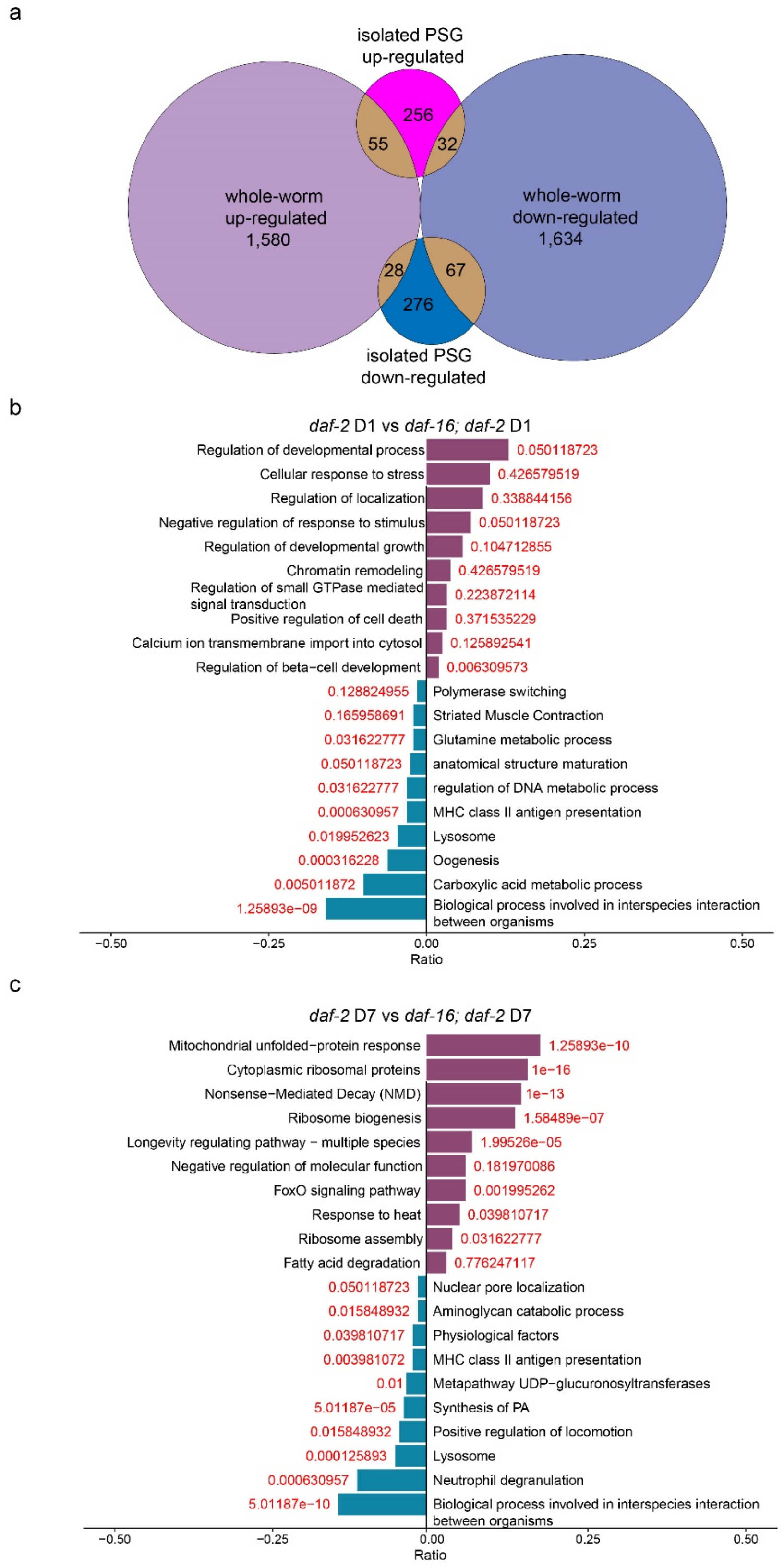

Supplementary Figure 2. Analysis of the PSG-specific DAF-16/FOXO transcriptomes, Related to Figure 1. a, Venn diagram showing the overlap between our PSG-specific and

previously reported whole-worm D1 DAF-16/FOXO transcriptomes[1, 2]. **b and c,** Pathway enrichment analysis of our D1 (**b**) and D7 (**c**) PSG-specific DAF-16/FOXO transcriptomes using Metascape with the whole genome as the background list. Up-regulated (purple) and down-regulated (blue) genes were analyzed separately.

a

C. elegans		*Drosophila*		Zebrafish		Human/Mouse	
**DSL**	**DOS**	**DSL**	**DOS**	**DSL**	**DOS**	**DSL**	**DOS**
LAG-2	DOS-1	Serrate	Serrate	DelataA	DelataA	Jagged1	Jagged1
APX-1	DOS-2	Delta	Delta	DelataB	DelataB	Jagged2	Jagged2
ARG-1	DOS-3	C901	C901	DelataC	DelataD	DLL1	DLL1
DSL-1	OSM-7			DelataD	DelataC	DLL3	DLL4
DSL-2	OSM-11					DLL4	DLK1
DSL-3							DLK2
DSL-4							
DSL-5							
DSL-6							
DSL-7							

b

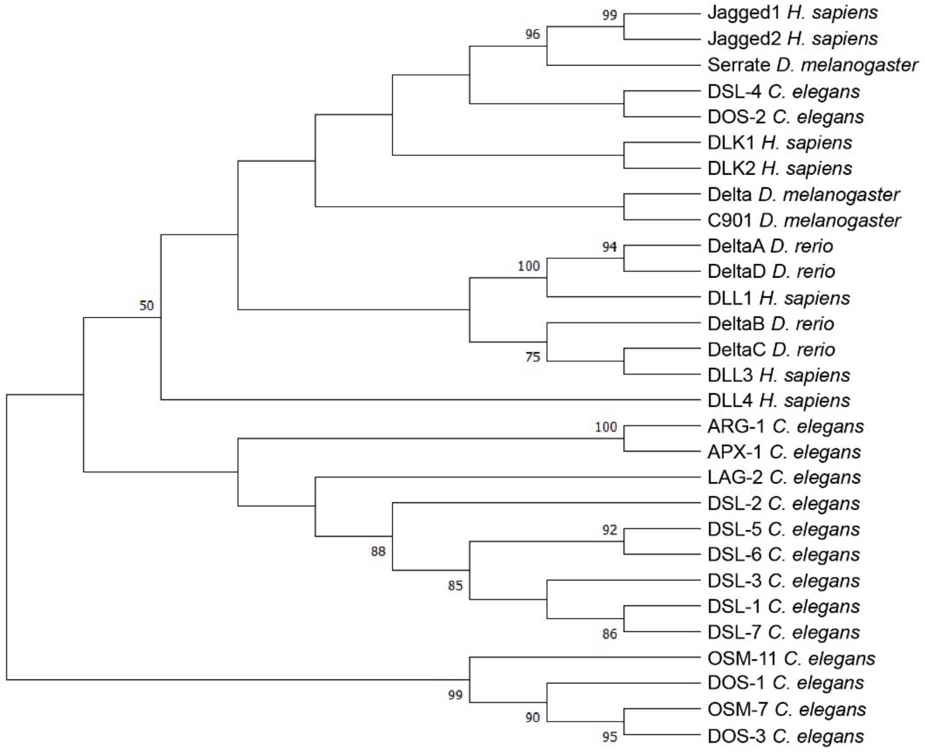

Supplementary Figure 3. Sequence analysis of Notch ligands, Related to Figure 1. a, Identification of DSL domain and/or DOS motif in a total of 29 Notch ligands from *C. elegans* (15), *Drosophila* (3), zebrafish (4), and human/mouse (7) using the Conserved Domain Database for DSL and the MyHits Pattern Search website (https://myhits.sib.swiss/cgi-bin/pattern_search) for DOS. **b**, Phylogenetic tree of the 29 Notch ligands. Protein sequences were aligned and scored using MUSCLE, and the phylogenetic tree was constructed using the maximum likelihood tree method.

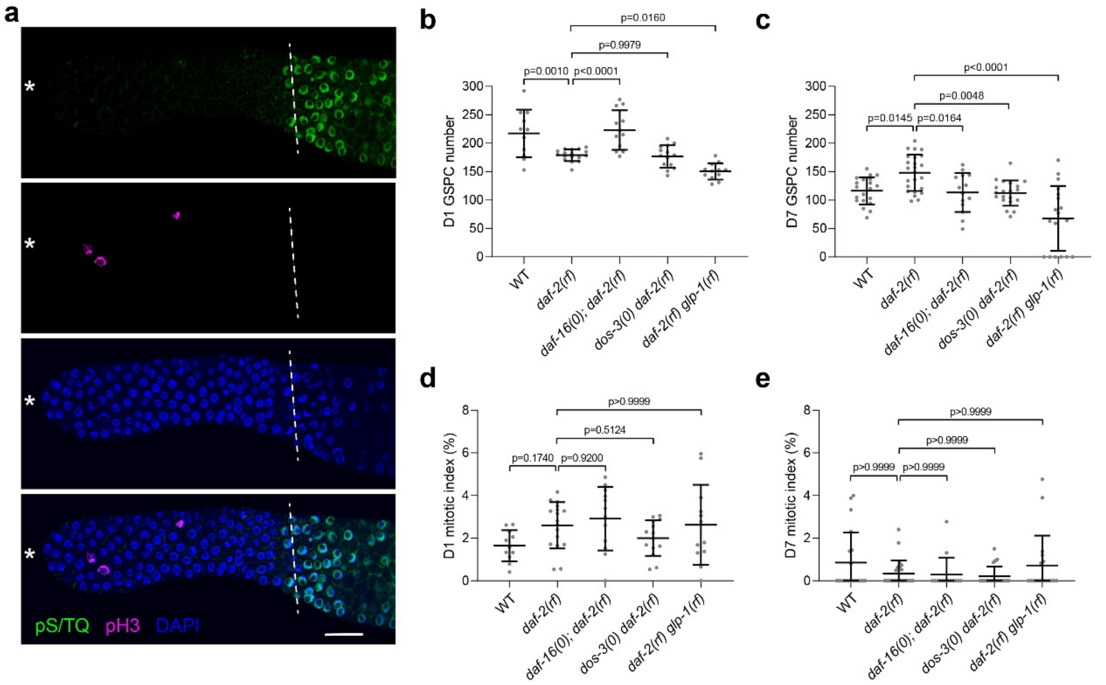

Supplementary Figure 4. Analysis of GSPCs using antibody staining, Related to Figure 3. a, A dissected gonad from a D1 wildtype animal stained with anti-pS/TQ antibody (green), anti-pH3 antibody (magenta), and DAPI (blue). Asterisk indicates the distal end of the germ line, and the white dashed line indicates the proximal border of the proliferative zone, defined as the first row of cells in which at least half of the nuclei are pS/TQ^{+}. Scale bar: 20 µm. **b and c,** Number of GSPCs per gonad arm in D1 (**b**) and D7 (**c**) wildtype, *daf-2(rf)*, *daf-16(0); daf-2(rf)*, *dos-3(0) daf-2(rf)*, and *daf-2(rf) glp-1(rf)* worms. **d and e,** Mitotic index of GSPCs in D1 (**d**) and D7 (**e**) wildtype, *daf-2(rf)*, *daf-16(0); daf-2(rf)*, *dos-3(0) daf-2(rf)*, and *daf-2(rf) glp-1(rf)* worms. Data are represented as mean ± SD with individual values shown as dots. n = 11, 17, 12, 14, 12 animals from left to right in (**b and d**); n = 20, 24, 14, 20, 18 animals in (**c and e**). P-values are calculated using one-way ANOVA and Dunnett's post hoc test. Source data are provided as a Source Data file.

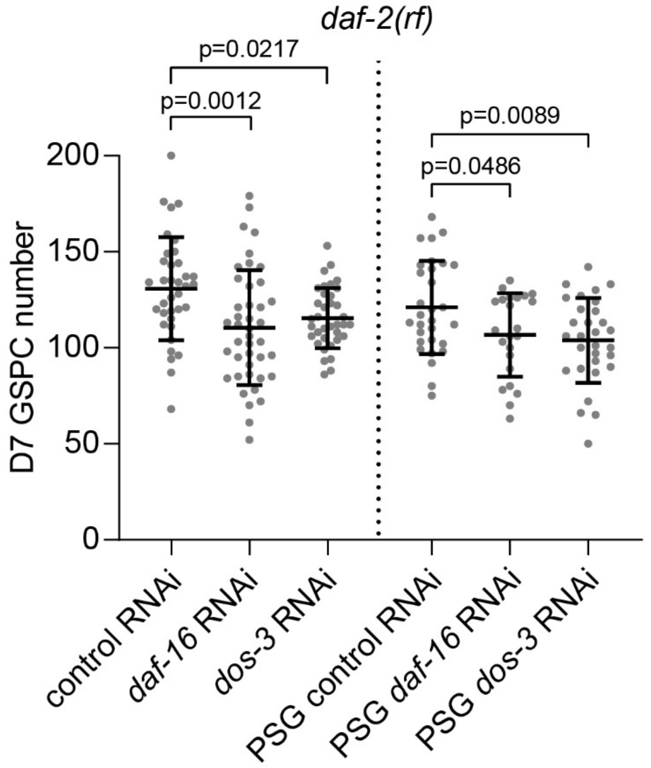

Supplementary Figure 5. *dos-3* is required in the adult PSG for the effect of reducing IIS on GSPC maintenance over time, Related to Figure 3. Number of GSPCs per gonad arm in D7 *daf-2(rf)* and *daf-2(rf); rde-1(0); Is[Pfos-1a::rde-1(+)]* animals treated with control, *daf-16* or *dos-3* RNAi from D1. Data are represented as mean ± SD with individual values shown as dots. n = 36, 42, 36, 30, 23, 30 animals from left to right. P-values are calculated using one-way ANOVA and LSD post hoc test. Source data are provided as a Source Data file.

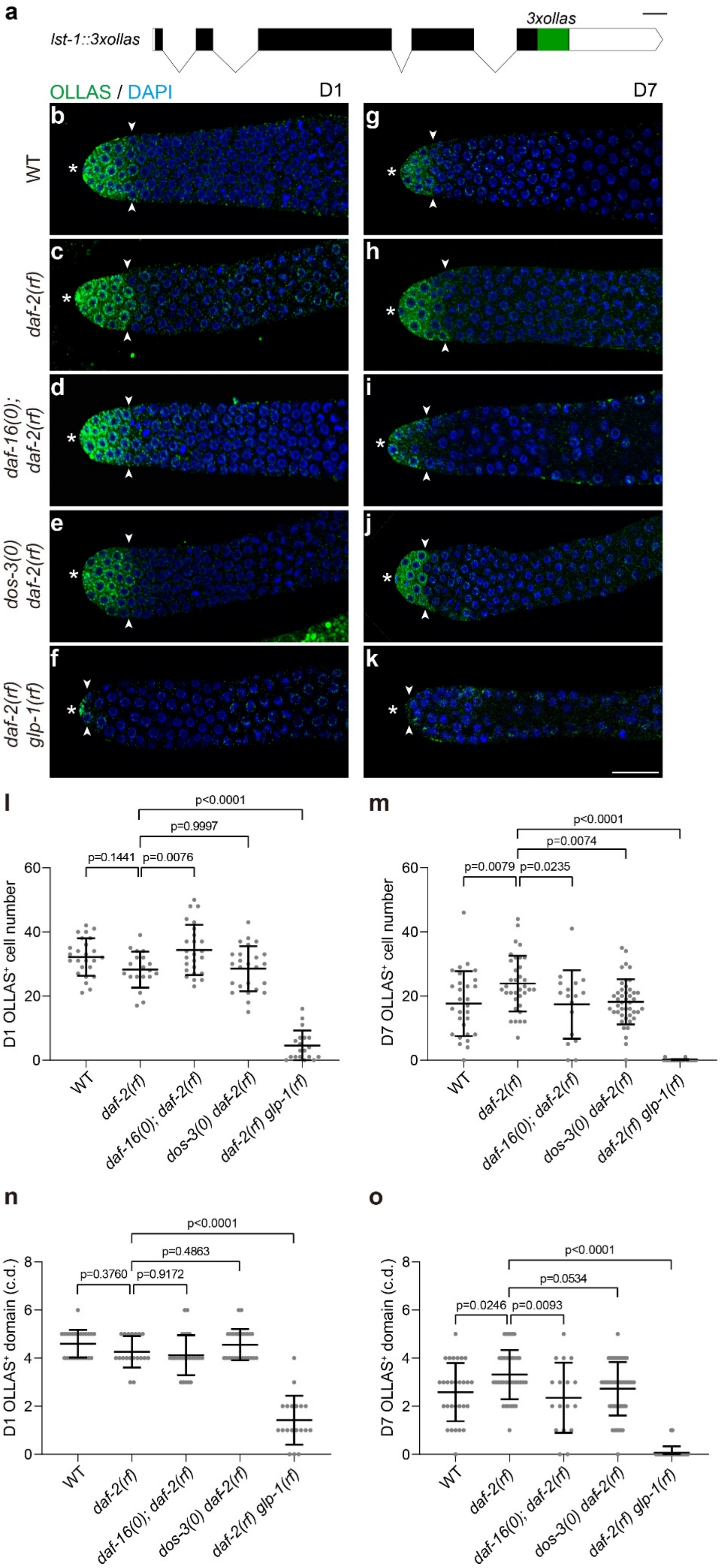

a
lst-1::3xollas
3xollas
OLLAS / DAPI
D1
D7
b
c
d
e
f
g
h
i
j
k
WT
daf-2(rf)
daf-16(0); daf-2(rf)
dos-3(0) daf-2(rf)
daf-2(rf) glp-1(rf)
l
m
n
o
D1 OLLAS+ cell number
D7 OLLAS+ cell number
D1 OLLAS+ domain (c.d.)
D7 OLLAS+ domain (c.d.)
p=0.1441
p=0.0076
p=0.9997
p<0.0001
p=0.0079
p=0.0235
p=0.0074
p=0.3760
p=0.9172
p=0.4863
p=0.0246
p=0.0093
p=0.0534

Supplementary Figure 6. Expression of *lst-1* in the distal germ line, Related to Figure 4. a, Schematic showing the CRISPR/Cas9 knock-in of *3xollas* at the C-terminus of *lst-1* endogenous locus. Graph generated using Exon-Intron Graphic Maker (http://www.wormweb.org/exonintron). Scale bar, 100 bp. **b-k,** Dissected gonads from D1 and D7 adults stained with anti-OLLAS antibody (green) and DAPI (blue). Asterisk indicates the distal end of the germ line, and the white arrowheads indicate the proximal border of the OLLAS staining. Scale bar: 20 μm. **b-f,** Representative germ lines of D1 wildtype (**b**), *daf-2(rf)* (**c**), *daf-16(0); daf-2(rf)* (**d**), *dos-3(0) daf-2(rf)* (**e**), and *daf-2(rf) glp-1(rf)* (**f**) animals expressing LST-1::OLLAS. **g-k,** Representative germ lines of D7 wildtype (**g**), *daf-2(rf)* (**h**), *daf-16(0); daf-2(rf)* (**i**), *dos-3(0) daf-2(rf)* (**j**), and *daf-2(rf) glp-1(rf)* (**k**) animals expressing LST-1::OLLAS. **l and m,** Number of $OLLAS^+$ cells per gonad arm in D1 (**l**) and D7 (**m**) wildtype, *daf-2(rf)*, *daf-16(0); daf-2(rf)*, *dos-3(0) daf-2(rf)*, and *daf-2(rf) glp-1(rf)* worms expressing LST-1::OLLAS. **n and o,** Size of $OLLAS^+$ domain measured in cell diameters (c.d.) in D1 (**n**) and D7 (**o**) wildtype, *daf-2(rf)*, *daf-16(0); daf-2(rf)*, *dos-3(0) daf-2(rf)*, and *daf-2(rf) glp-1(rf)* worms expressing LST-1::OLLAS. Data are represented as mean ± SD with individual values shown as dots. n = 25, 19, 25, 25, 19 animals from left to right in (**1 and n**); n = 29, 35, 17, 44, 28 animals in (**m and o**). P-values are calculated using one-way ANOVA and Dunnett's post hoc test. Source data are provided as a Source Data file.

Supplementary Table 1. Strains.

Strain	Genotype	Source
N2	wild type	Caenorhabditis Genetics Center
CB1370	*daf-2(e1370)*	Caenorhabditis Genetics Center
GC1332	*daf-16(mu86); daf-2(e1370)*	Jane Hubbard[3]
PHX1236	*unc-119(ed3); lsaSi1[Pfos-1a::gfp::unc-54 3'UTR + unc-119(+)] II*	SunyBiotech
QIN7	*lsaSi1[Pfos-1a::gfp::unc-54 3'UTR + unc-119(+)]; daf-2(e1370)*	This study
QIN9	*daf-16(mu86); lsaSi1[Pfos-1a::gfp::unc-54 3'UTR + unc-119(+)]; daf-2(e1370)*	This study
QIN50	*lsaEx13[Pdos-3(wt)::gfp::unc-54 3'UTR; Pmyo-3::mcherry]*	This study
QIN51	*lsaEx14[Pdos-3(wt)::gfp::unc-54 3'UTR; Pmyo-3::mcherry]*	This study
QIN53	*lsaEx16[Pdos-3(mut)::gfp::unc-54 3'UTR; Pmyo-3::mcherry]*	This study
QIN54	*lsaEx17[Pdos-3(mut)::gfp::unc-54 3'UTR; Pmyo-3::mcherry]*	This study
QIN55	*lsaEx18[Pdos-3(mut)::gfp::unc-54 3'UTR; Pmyo-3::mcherry]*	This study
QIN57	*daf-2(e1370); lsaEx13[Pdos-3(wt)::gfp::unc-54 3'UTR; Pmyo-3::mcherry]*	This study
QIN58	*daf-2(e1370); lsaEx14[Pdos-3(wt)::gfp::unc-54 3'UTR; Pmyo-3::mcherry]*	This study
QIN61	*daf-2(e1370); lsaEx16[Pdos-3(mut)::gfp::unc-54 3'UTR; Pmyo-3::mcherry]*	This study
QIN62	*daf-2(e1370); lsaEx17[Pdos-3(mut)::gfp::unc-54 3'UTR; Pmyo-3::mcherry]*	This study
QIN63	*daf-2(e1370); lsaEx18[Pdos-3(mut)::gfp::unc-54 3'UTR; Pmyo-3::mcherry]*	This study
GC832	*glp-1(e2141)*	Jane Hubbard[3, 4]
QIN56	*daf-2(e1370) glp-1(e2141)*	This study
PHX5125	*dos-3(syb5125)*	SunyBiotech
QIN67	*dos-3(syb5125) daf-2(e1370)*	This study
GC1352	*daf-2(e1370); rde-1(ne219); qyIs102[Pfos-1a::rde-1(genomic) + myo-2::yfp + unc-119(+)]*	Jane Hubbard[3, 5]
PHX6093	*3xollas::sygl-1*	SunyBiotech
QIN74	*3xollas::sygl-1; daf-2(e1370)*	This study
QIN88	*daf-16(mu86) 3xollas::sygl-1; daf-2(e1370)*	This study

QIN75	*3xollas::sygl-1; dos-3(syb5125) daf-2(e1370)*	This study
QIN78	*3xollas::sygl-1; daf-2(e1370) glp-1(e2141)*	This study
PHX5757	*lst-1::3xollas*	SunyBiotech
QIN76	*lst-1::3xollas; daf-2(e1370)*	This study
QIN89	*lst-1::3xollas daf-16(mu86); daf-2(e1370)*	This study
QIN77	*lst-1::3xollas; dos-3(syb5125) daf-2(e1370)*	This study
QIN80	*lst-1::3xollas; daf-2(e1370) glp-1(e2141)*	This study
QIN64	*lsaEx19[Phsp-16.2::dos-3 cDNA::unc-54 3'UTR; Pmyo-2::gfp]*	This study
QIN65	*lsaEx20[Phsp-16.2::dos-3 cDNA::unc-54 3'UTR; Pmyo-2::gfp]*	This study
QIN97	*daf-16(mu86); daf-2(e1370); lsaEx19[Phsp-16.2::dos-3 cDNA::unc-54 3'UTR; Pmyo-2::gfp]*	This study
QIN98	*daf-16(mu86); daf-2(e1370); lsaEx20[Phsp-16.2::dos-3 cDNA::unc-54 3'UTR; Pmyo-2::gfp]*	This study
JK1277	*lag-2(q420)*	Caenorhabditis Genetics Center
QIN99	*lag-2(q420); lsaEx19[Phsp-16.2::dos-3 cDNA::unc-54 3'UTR; Pmyo-2::gfp]*	This study
QIN100	*lag-2(q420); lsaEx20[Phsp-16.2::dos-3 cDNA::unc-54 3'UTR; Pmyo-2::gfp]*	This study
QIN101	*glp-1(e2141); lsaEx19[Phsp-16.2::dos-3 cDNA::unc-54 3'UTR; Pmyo-2::gfp]*	This study
QIN102	*glp-1(e2141); lsaEx20[Phsp-16.2::dos-3 cDNA::unc-54 3'UTR; Pmyo-2::gfp]*	This study
QIN93	*3xollas::sygl-1; lsaEx19[Phsp-16.2::dos-3 cDNA::unc-54 3'UTR; Pmyo-2::gfp]*	This study
QIN94	*3xollas::sygl-1; lsaEx20[Phsp-16.2::dos-3 cDNA::unc-54 3'UTR; Pmyo-2::gfp]*	This study
QIN96	*lst-1::3xollas; lsaEx19[Phsp-16.2::dos-3 cDNA::unc-54 3'UTR; Pmyo-2::gfp]*	This study
QIN95	*lst-1::3xollas; lsaEx20[Phsp-16.2::dos-3 cDNA::unc-54 3'UTR; Pmyo-2::gfp]*	This study
QIN84	*lsaEx25[Phsp-16.2::DLK1 cDNA::unc-54 3'UTR; Pmyo-2::gfp]*	This study
QIN85	*lsaEx26[Phsp-16.2::DLK1 cDNA::unc-54 3'UTR; Pmyo-2::gfp]*	This study

Supplementary References

1. Murphy, C. T. *et al.* Genes that act downstream of DAF-16 to influence the lifespan of *Caenorhabditis elegans*. *Nature* **424**, 277-284 (2003).
2. Tepper, R. G. *et al.* PQM-1 complements DAF-16 as a key transcriptional regulator of DAF-2-mediated development and longevity. *Cell* **154**, 676-690 (2013).
3. Qin, Z. & Hubbard, E. J. A. Non-autonomous DAF-16/FOXO activity antagonizes age-related loss of *C. elegans* germline stem/progenitor cells. *Nat. Commun.* **6**, 7107 (2015).
4. Dalfó, D., Priess, J. R., Schnabel, R. & Hubbard, E. J. A. *The Worm Breeder's Gazette* http://wbg.wormbook.org/2010/12/06/glp-1e2141-sequence-correction/ (2010).
5. Hagedorn, E. J. *et al.* Integrin acts upstream of netrin signaling to regulate formation of the anchor cell's invasive membrane in *C. elegans*. *Developmental Cell* **17**, 187-198 (2009).
