# Supplementary material for: DOS-3 mediates cell-non-autonomous DAF-16/FOXO activity in antagonizing age-related loss of C. elegans germline stem/progenitor cells
Source: Nat Commun. 2024 Jun 8;15:4904. doi: 10.1038/s41467-024-49318-6 (PMC11162419; doi:10.1038/s41467-024-49318-6)
Supplement: Supplementary file 3 — Reporting Summary [file 41467_2024_49318_MOESM3_ESM.pdf]

nature portfolio

Corresponding author(s): Jun Xiang, Jialin C. Zheng, and Zhao Qin

Last updated by author(s): May 6, 2024

Reporting Summary

Nature Portfolio wishes to improve the reproducibility of the work that we publish. This form provides structure for consistency and transparency in reporting. For further information on Nature Portfolio policies, see our [Editorial Policies] and the [Editorial Policy Checklist].

Statistics

For all statistical analyses, confirm that the following items are present in the figure legend, table legend, main text, or Methods section.

n/a	Confirmed	
☐	☒	The exact sample size (n) for each experimental group/condition, given as a discrete number and unit of measurement
☒	☐	A statement on whether measurements were taken from distinct samples or whether the same sample was measured repeatedly
☐	☒	The statistical test(s) used AND whether they are one- or two-sided<br>*Only common tests should be described solely by name; describe more complex techniques in the Methods section.*
☒	☐	A description of all covariates tested
☐	☒	A description of any assumptions or corrections, such as tests of normality and adjustment for multiple comparisons
☐	☒	A full description of the statistical parameters including central tendency (e.g. means) or other basic estimates (e.g. regression coefficient) AND variation (e.g. standard deviation) or associated estimates of uncertainty (e.g. confidence intervals)
☐	☒	For null hypothesis testing, the test statistic (e.g. F, t, r) with confidence intervals, effect sizes, degrees of freedom and P value noted<br>*Give P values as exact values whenever suitable.*
☒	☐	For Bayesian analysis, information on the choice of priors and Markov chain Monte Carlo settings
☒	☐	For hierarchical and complex designs, identification of the appropriate level for tests and full reporting of outcomes
☒	☐	Estimates of effect sizes (e.g. Cohen's d, Pearson's r), indicating how they were calculated

Our web collection on statistics for biologists contains articles on many of the points above.

Software and code

Policy information about availability of computer code

Data collection	FACS: FACSAriaII cell sorter (BD Biosciences) using DIVA8.0.1 software (BD Biosciences);<br>RNA-seq: Bioanalyzer 2100 system (Agilent Technologies) and Novaseq 6000 platform (Illumina);<br>Images: Zeiss Imager M1 equipped with an Apotome Axioimager (Carl Zeiss) and LSM880 confocal laser scanning microscope (Carl Zeiss).
Data analysis	FACS: Flowjo v10;<br>RNA-seq: Hisat2 v2.0.5, featureCounts v1.5.0-p3, and DESeq2 R package (1.20.0);<br>Images: Zeiss Zen Imaging Softwares (Zen 3.4 and Zen 2.3 pro) and Fiji (ImageJ);<br>Protein sequence alignment and phylogenetic analysis: MEGA 11 and Jalview;<br>Statistical analyses: GraphPad Prism 8.

For manuscripts utilizing custom algorithms or software that are central to the research but not yet described in published literature, software must be made available to editors and reviewers. We strongly encourage code deposition in a community repository (e.g. GitHub). See the Nature Portfolio guidelines for submitting code & software for further information.

Data

Policy information about availability of data

All manuscripts must include a data availability statement. This statement should provide the following information, where applicable:
- Accession codes, unique identifiers, or web links for publicly available datasets
- A description of any restrictions on data availability
- For clinical datasets or third party data, please ensure that the statement adheres to our policy

Source data are provided with this paper. The RNA-seq data generated in this study have been deposited in the Gene Expression Omnibus (GEO) database under accession code GSE243994 [https://www.ncbi.nlm.nih.gov/geo/query/acc.cgi?acc=GSE243994].

Research involving human participants, their data, or biological material

Policy information about studies with human participants or human data. See also policy information about sex, gender (identity/presentation), and sexual orientation and race, ethnicity and racism.

Reporting on sex and gender	N/A
Reporting on race, ethnicity, or other socially relevant groupings	N/A
Population characteristics	N/A
Recruitment	N/A
Ethics oversight	N/A

Note that full information on the approval of the study protocol must also be provided in the manuscript.

Field-specific reporting

Please select the one below that is the best fit for your research. If you are not sure, read the appropriate sections before making your selection.

☒ Life sciences ☐ Behavioural & social sciences ☐ Ecological, evolutionary & environmental sciences

For a reference copy of the document with all sections, see nature.com/documents/nr-reporting-summary-flat.pdf

Life sciences study design

All studies must disclose on these points even when the disclosure is negative.

Sample size	Sample sizes were based on previously published studies by our laboratory that used comparable methodology (Qin and Hubbard 2015, Nature Communications; Liang et al. 2023, iScience). We generally use sample sizes > 10 animals unless there is a difficulty in obtaining so many animals, e.g., transgenic animals carrying extrachromosomal arrays with low transmission rate.
Data exclusions	No data were excluded.
Replication	Data are representative of at least three biological replicates and all attempts at replication were successful.
Randomization	Animals were allocated randomly into experimental groups.
Blinding	Blinding was not required for analyses performed in this study. Experiments that used comparable methodology were previously published by our laboratory (Qin and Hubbard 2015, Nature Communications; Liang et al. 2023, iScience).

Reporting for specific materials, systems and methods

We require information from authors about some types of materials, experimental systems and methods used in many studies. Here, indicate whether each material, system or method listed is relevant to your study. If you are not sure if a list item applies to your research, read the appropriate section before selecting a response.

Materials & experimental systems

n/a	Involved in the study
	☒ Antibodies
☒	☐ Eukaryotic cell lines
☒	☐ Palaeontology and archaeology
	☒ Animals and other organisms
☒	☐ Clinical data
☒	☐ Dual use research of concern
☒	☐ Plants

Methods

n/a	Involved in the study
☒	☐ ChIP-seq
	☒ Flow cytometry
☒	☐ MRI-based neuroimaging

Antibodies

Antibodies used	rabbit anti-pS/TQ (Cell Signaling Technology, cat#: 6966, lot: 8, 1:500, RRID: AB_10949894); mouse anti-pH3 (Cell Signaling Technology, cat#: 9706, lot: 10, 1:150, RRID: AB_331748); rat anti-OLLAS (Novus Biologicals, cat#: NBP1-06713, lot: F16, 1:2000, RRID: AB_1625979); Alexa 488 goat anti-rabbit IgG (Jackson, cat#: 111-545-003, lot: 168597, 1:600, RRID: AB_2338046); Alexa 594 goat anti-mouse IgG (Jackson, cat#: 115-585-003, lot: 168330, 1:600, RRID: AB_2338871); Alexa 488 donkey anti-rat IgG (Invitrogen, cat#: A-21208, lot: A21208, 1:500, RRID: AB_2535794).
Validation	The primary antibodies used in this study were extensively validated and had publications tested in C. elegans. References and manufacturer validation can be found through the following links: https://www.cellsignal.cn/products/primary-antibodies/phospho-atm-atr-substrate-motif-ps-pt-qg-multimab-rabbit-mab-mix/6966 (anti-pS/TQ); https://www.cellsignal.cn/products/primary-antibodies/phospho-histone-h3-ser10-6g3-mouse-mab/9706 (anti-pH3); https://www.novusbio.com/products/ollas-epitope-tag-antibody-l2_nbp1-06713#datasheet (anti-OLLAS).

Animals and other research organisms

Policy information about studies involving animals; ARRIVE guidelines recommended for reporting animal research, and Sex and Gender in Research

Laboratory animals	All C. elegans strains used in this study are listed in Supplementary Table 1. Adult hermaphrodites were examined on D1 or on D7.
Wild animals	No wild animals were used in this study.
Reporting on sex	Hermaphrodites were used in all analyses performed in this study.
Field-collected samples	No samples collected from the field were used in this study.
Ethics oversight	No ethical approval or guidance was needed.

Note that full information on the approval of the study protocol must also be provided in the manuscript.

Flow Cytometry

Plots

Confirm that:

☒ The axis labels state the marker and fluorochrome used (e.g. CD4-FITC).

☒ The axis scales are clearly visible. Include numbers along axes only for bottom left plot of group (a 'group' is an analysis of identical markers).

☒ All plots are contour plots with outliers or pseudocolor plots.

☒ A numerical value for number of cells or percentage (with statistics) is provided.

Methodology

Sample preparation	D1 and D7 daf-2(rf) and daf-16(0); daf-2(rf) worms labeled with Pfos-1a::gfp were used for PSG cell isolation. For cell dissociation, worms were treated with proteinases and mechanically disrupted. After dissociation, GFP+ cells were immediately isolated by FACS. More detailed information regarding tissue processing is described in “Methods-FACS isolation of adult PSG cells”.
Instrument	BD FACSAriaII cell sorter
Software	Flowjo v10
Cell population abundance	The abundance of the GFP+ cell population ranged from 3-8%.

Gating strategy

The cell suspension was first gated using FSC-A/SSC-A to exclude debris and dead cells, then cell clumps were removed using FSC-W/FSC-H. Lastly, GFP fluorescence was verified and analyzed using FITC-A/SSC-A.

☒ Tick this box to confirm that a figure exemplifying the gating strategy is provided in the Supplementary Information.
